# Phenotype clustering in health care: A narrative review for clinicians

**DOI:** 10.3389/frai.2022.842306

**Published:** 2022-08-12

**Authors:** Tyler J. Loftus, Benjamin Shickel, Jeremy A. Balch, Patrick J. Tighe, Kenneth L. Abbott, Brian Fazzone, Erik M. Anderson, Jared Rozowsky, Tezcan Ozrazgat-Baslanti, Yuanfang Ren, Scott A. Berceli, William R. Hogan, Philip A. Efron, J. Randall Moorman, Parisa Rashidi, Gilbert R. Upchurch, Azra Bihorac

**Affiliations:** ^1^Department of Surgery, University of Florida Health, Gainesville, FL, United States; ^2^Precision and Intelligent Systems in Medicine (PrismaP), University of Florida, Gainesville, FL, United States; ^3^Intelligent Critical Care Center, University of Florida, Gainesville, FL, United States; ^4^Department of Medicine, University of Florida Health, Gainesville, FL, United States; ^5^Departments of Anesthesiology, Orthopedics, and Information Systems/Operations Management, University of Florida Health, Gainesville, FL, United States; ^6^Department of Health Outcomes and Biomedical Informatics, College of Medicine, University of Florida, Gainesville, FL, United States; ^7^Department of Medicine, University of Virginia, Charlottesville, VA, United States; ^8^Departments of Biomedical Engineering, Computer and Information Science and Engineering, and Electrical and Computer Engineering, University of Florida, Gainesville, FL, United States

**Keywords:** machine learning, artificial intelligence, cluster, endotype, endotyping

## Abstract

Human pathophysiology is occasionally too complex for unaided hypothetical-deductive reasoning and the isolated application of additive or linear statistical methods. Clustering algorithms use input data patterns and distributions to form groups of similar patients or diseases that share distinct properties. Although clinicians frequently perform tasks that may be enhanced by clustering, few receive formal training and clinician-centered literature in clustering is sparse. To add value to clinical care and research, optimal clustering practices require a thorough understanding of how to process and optimize data, select features, weigh strengths and weaknesses of different clustering methods, select the optimal clustering method, and apply clustering methods to solve problems. These concepts and our suggestions for implementing them are described in this narrative review of published literature. All clustering methods share the weakness of finding potential clusters even when natural clusters do not exist, underscoring the importance of applying data-driven techniques as well as clinical and statistical expertise to clustering analyses. When applied properly, patient and disease phenotype clustering can reveal obscured associations that can help clinicians understand disease pathophysiology, predict treatment response, and identify patients for clinical trial enrollment.

## Introduction

Human pathophysiology is extraordinarily complex. There are ~68,000 diagnostic codes in the 10th revision of the International Statistical Classification of Diseases (ICD) system, and more than 10,000 Current Procedural Terminology (CPT) treatment codes. Individual patients may have any combination of diagnoses, treatments, and treatment responses that are influenced by any combination of behavioral, social, and genetic determinants of health. Unsurprisingly, clinical decision-making based solely on hypothetical-deductive reasoning is error-prone and patient outcomes vary substantially (Wolf et al., 1985; Kirch and Schafii, 1996; Graber et al., 2005; Bekker, 2006; Dijksterhuis et al., 2006).

The inherent weaknesses in hypothetical-deductive reasoning for diagnosing and treating complex pathophysiology are addressed partially by clinical decision support systems (Hunt et al., 1998). The algorithms underlying decision support influence their efficacy. For example, efforts to represent the complex pathophysiology of frailty or sepsis using rule-based, additive or linear statistical methods have yielded suboptimal results, though linear models can also function effectively as data mining techniques (Lipsitz and Goldberger, 1992; Singer et al., 2016; Bertsimas et al., 2018; Loftus et al., 2019). In contrast, machine learning techniques, like clustering, learn from data (Schwartz et al., 1987; Hashimoto et al., 2018). Patient and disease phenotype clustering can elucidate pathophysiology, can predict treatment response, and has the potential to augment clinical trial enrollment (Calfee et al., 2014, 2018; Famous et al., 2017; Sinha et al., 2018; Seymour et al., 2019). Although clinicians perform these tasks frequently in routine, clinical practice (e.g., establishing differentials) and in research, few receive formal training necessary to apply clustering methods, and clinician-centered literature in clustering is sparse.

This narrative review of published literature endeavors to impart understanding of phenotype clustering in health care for clinicians by reviewing basic data processing and optimization steps; describing the concepts, strengths, and weaknesses of prominent clustering methods; suggesting a framework for choosing a clustering method; noting instances in which cluster phenotyping can elucidate pathophysiology and predict treatment response; and identifying opportunities to enhance clinical trial enrollment.

## Overview of phenotype clustering in health care

**Figure 1:**
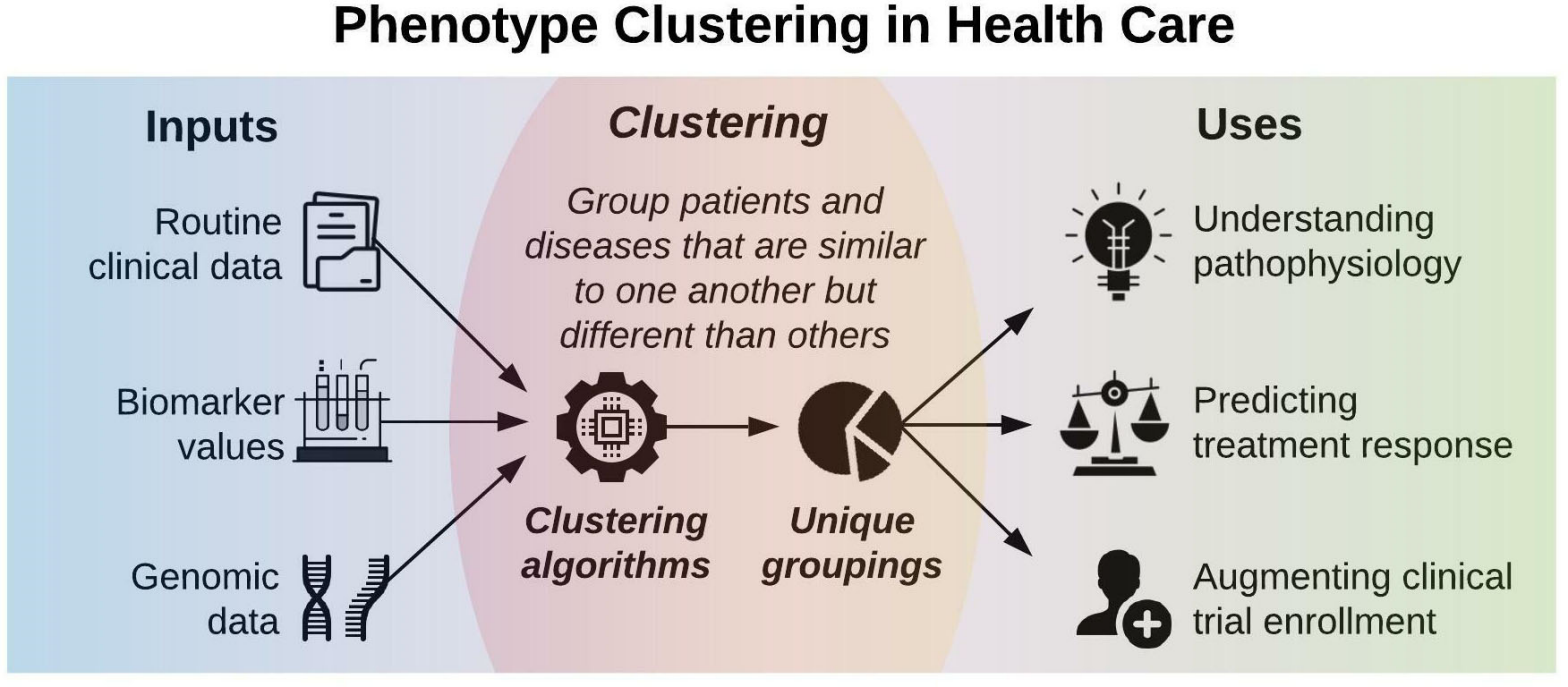
Phenotype clustering in health care applies clustering algorithms to clinical data, biomarkers, or genomic data to form unique groupings that can elucidate pathophysiology, predict treatment response, or augment clinical trial enrollment.

**Figure 2:**
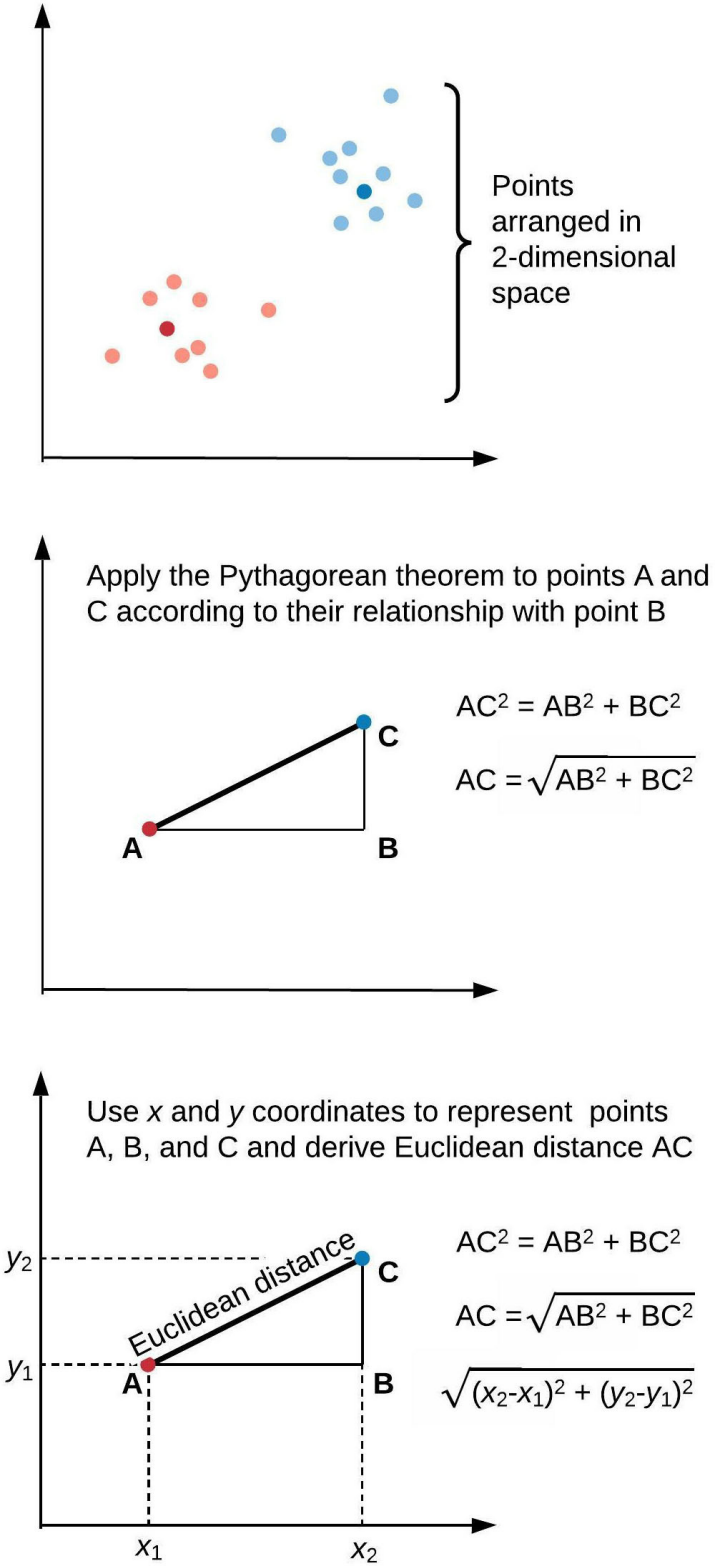
Similarity of elements in clustering algorithms is inversely proportional to distance. This is often derived by applying the Pythagorean theorem to calculate Euclidean distance. We illustrate this approach in two-dimensional space, though similar calculations apply for data points of arbitrary dimensionality.

Figure 1 illustrates a framework for phenotype clustering in health care. Clustering algorithms use input data patterns and distributions to form groups of patients or diseases that are similar to one another and different than others. Common input features include clinical data, biomarkers, and genomic data. There are six major methods for clustering, each with unique conceptual approaches, similarity metrics, and grouping techniques. Each algorithm has unique strengths and weaknesses depending on its specific application, but they all apply the same high-level methodology. First, the notion of similarity between two data points must be defined. This is often done by determining geometric distances between points, such that adjacent objects share similar characteristics, while objects with the greatest distances between them have the least similarity. This is commonly performed by calculating the Euclidean distance between two points, as illustrated in Figure 2.

Here, Euclidean distance is illustrated in two dimensions (*x* and *y*), though the same approach can be extended to any number of dimensions. Next, objects are grouped with other objects according to similarity relationships, forming clusters. Every dataset object is assigned membership to a group. This approach returns clusters even if there are no natural groups in the data. Finally, since clustering is often performed for data exploration or pattern discovery without establishing ground truth, validation often requires deriving or predicting cluster labels in a separate dataset and comparing cluster characteristics between development and validation cohorts. The fundamental concepts, strengths, and weaknesses of six major clustering methods are summarized in Table 1 and described in greater detail in the “Clustering methods” section.

**Table 1:** Summary of clustering methods.

**Method**	**Examples**	**Concept**	**Strengths**	Weaknesses^*^
Centroid-based or partitional clustering	*K*-means, *k*-medians	Minimize the distance between points within a cluster while maximizing the distance between cluster centroids	Simple implementation and interpretation	Number of clusters must be assigned *a priori*; sensitivity to outliers
Centroid-based variation: fuzzy clustering	Fuzzy c-means, rough or soft k-means	Points are assigned to one or more clusters based on membership coefficients representing similarity to other points in each cluster	Useful for datasets and applications with substantial overlap like image segmentation or genomic clustering	Number of clusters must be assigned *a priori*; slow convergence for large datasets; sensitivity to outliers
Hierarchical clustering	DIANA, AGNES	Generate a dendrogram using distance metrics and then cut the dendrogram to group its components	Obviates defining the number of clusters *a priori*; dendrograms are easy to interpret	Cumbersome for large datasets; sensitivity to outliers
Distribution-based clustering	Gaussian mixed models, DBCLASD	Points are assigned to clusters with similar probability distributions for metrics like mean and variance	Flexible, adapts to inherent distributions of the data, if present	Tends to overfit noisy data, complex algorithm runs slowly on large datasets
Density-based clustering	DBSCAN, Mean shift, OPTICS	Clusters are identified as the densest region in a data space, separated from other clusters by low-density areas	Adapts to non-linear data; obviates spatial and shape constraints of the clusters; insensitivity to outliers	Performs poorly with sparse data; sensitive to hyperparameters; complex algorithm runs slowly on large datasets
Supervised or constraint-based clustering	Random forest, gradient boosting, deep learning	Certain properties of the clustering result are defined *a priori*, like cluster number, size, dimensions, or elements	Incorporates prior knowledge of biology; generates a perfect decision boundary	Greater risk of overfitting compared with unsupervised methods
Spectral or graph-based clustering	STING, CLIQUE	Use a standard (e.g., *k*-means) clustering method on special vectors (eigenvectors) or densities within a matrix that represents a graph	Effective for high-dimensional spectral data that contains substantial noise and outliers	Cumbersome for large graphs, interpretation requires understanding of vector spaces and linear transformation

*DIANA, Divisive ANAlysis; AGNES, AGlomerative NESting; DBCLASD, Distribution-Based Clustering of LArge Spatial Databases; DBSCAN, density-based spatial clustering of applications with noise; OPTICS, ordering points to identify the clustering structure; STING, statistical information grid; CLIQUE, Clustering In QUEst*.

* *All clustering methods share the weakness of finding clusters even when natural clusters do not exist*.

## Clustering input data processing, and optimization

The raw clinical data that is typically available to clinicians often cannot be applied directly to clustering algorithms. This section describes data processing and optimization steps that are intended to produce optimal clustering results, primarily using terms and descriptions that are familiar to clinicians. For more technical descriptions of data processing and optimization steps, interested readers are referred to more technical work by Ankerst et al. (1999) and Yu et al. (2015).

### Handling outliers

Clustering-based phenotyping in health care has been performed using routine clinical data, biomarker values, and genomic data, each of which often contain outliers (Eisen et al., 1998; Seymour et al., 2019). If the clustering method is sensitive to outlier values, as described below, outliers may be clipped at predetermined percentiles (e.g., removing the top and bottom 1% of all values or values more than 3 times the interquartile range beyond the 25th or 75th percentile), but this approach risks losing important information from true values that deviate substantially from the rest of the data. Therefore, we recommend handling outliers on a variable-by-variable basis according to statistical knowledge and clinical expertise.

### Handling missing data

Data missing at random should be imputed (replaced with a substituted value), ideally with a method that accounts for statistical uncertainty in the imputations, such as MICE (multiple imputation by chained equations; Van Buuren et al., 1999). When data are missing not at random (e.g., bilirubin levels are missing because there was no clinical concern for hepatic dysfunction), there is currently no consensus regarding how to handle the missing data appropriately; it may be favorable to use binary missingness indicators (indicators of whether the variable is missing) or other techniques that preserve potentially informative missingness patterns (Jakobsen et al., 2017).

### Scaling data

When continuous variables within the same dataset have different ranges or magnitudes of change, those with wider distributions will dominate cluster assignments. For example, one may wish to include both serum creatinine and platelet count values as clustering input variables. Two patients with serum creatinine values of 1.0 vs. 4.0 mg/dL have substantially different renal function; two patients with platelet counts of 101 vs. 104 x 10^9^/L have no meaningful difference in platelet counts. This issue of scale is addressed by normalizing the data (transforming each variable into a common range such as 0–1). On a scale from 0 to 1, serum creatinine values of 1.0 and 4.0 mg/dL might be represented as 0.30 and 0.80, respectively, while platelet counts of 100 vs. 104 x 10^9^/L might be represented as 0.33 and 0.34, respectively. In this case, the normalized values more accurately represent differences in creatinine values and similarity between platelet values. Other normalization techniques calculate a z-score for each value, scaling the data by mean and standard deviation.

### Handling categorical variables

Common distance metrics like Euclidean and Manhattan distance apply only to continuous variables, but many potentially important clinical variables (e.g., sex) are categorical, with no ordinal mathematical interpretation. There are several methods for addressing this challenge. Most simply, clustering may be performed on continuous variables only, ignoring categorical variables. Categorical variables can be converted into continuous variables by several methods (e.g., *n* binary features or value difference metrics; Grabczewski and Jankowski, 2003). An alternative distance metric can be used, like Gower distance, which calculates distance between entities composed of both continuous and categorical variables. Finally, one can apply *k*-modes clustering, which defines clusters by matched categories (Huang, 1998).

### Performing data transformation

Clustering on continuous variables tends to be very effective on normally distributed data. Therefore, it is often advantageous to perform natural log or power transformations on non-normal variables prior to clustering (Seymour et al., 2019). To preserve the original data distributions, one may perform density-based clustering, which makes no assumptions about data distributions (this is described below in the “Clustering methods” section).

### Performing feature selection

When clustering a dataset containing many features (variables), some features have greater importance in cluster assignments, while others introduce distracting noise. To mitigate the impact of noisy features, one may select a subset of features on which to perform clustering. In some cases, there may be a clinical precedent for selecting features. For example, the sequential organ failure assessment (SOFA) score is a well-validated metric of organ dysfunction. To identify organ dysfunction clusters, one may simply select variables used to calculate SOFA scores. When there is no such clinical precedent, feature selection can be performed by dimensionality reduction techniques such as principal component analysis (PCA) to derive underlying, lower-dimensional data signatures from combinations or mixtures of complex, high-dimensional data. Alternatively, one may perform clustering on all available features, rank their importance in determining cluster assignments, and then select only the most important features for subsequent analyses. Decisions involving feature selection methods and the inclusion or exclusion of specific features must be carefully considered since feature selection can inadvertently eliminate meaningful features along with noisy features, thereby biasing clustering results.

## Types of clustering algorithms

This section describes clustering methods that have been applied in healthcare, and corresponds to a summary of clustering algorithms in Table 1. For a robust description of other important clustering methods that have not yet been applied in healthcare, such as possibilistic clustering, interested readers are referred to work by Krishnapuram and Keller (1993), Pal et al. (2005), Antoine et al. (2018), and Koutsibella and Koutroumbas (2020).

### Centroid-based clustering

Centroid-based methods, sometimes called partitioning methods, minimize distance between points within a cluster while maximizing the distance between cluster centroids, or the geometric center of each cluster. *K*-means clustering is the most prominent example of centroid-based clustering. First, one chooses a number of clusters, *k*. The algorithm randomly selects *k* data points as centroids. Next, the algorithm calculates similarity between each point and each centroid, as described in the “Overview of phenotype clustering in health care” section. Each point is grouped with its nearest centroid. Then, each centroid's position is updated by calculating the geometric mean among its constituent data points, and cluster memberships are again reassigned based on centroid distances. The process is repeated until centroid positions and cluster assignments remain constant. Centroid-based clustering has relatively simple implementation and interpretation, which likely contributes to its popularity in health care applications. The optimal number of clusters is usually unknown in advance, and is found instead *via* trial-and-error experimentation; different *k*-values are ranked by within-cluster similarity and pairs of clusters are compared to determine whether they should be merged into one cluster (Altman and Krzywinski, 2017). Centroid-based clustering is sensitive to outliers; this limitation can be leveraged for outlier detection (Nowak-Brzezinska and Lazarz, 2021). When computational power allows, experimentation with different *k*-values and clustering iterations can provide unique advantages for exploratory classifications. For example, *k*-means clustering was used to build 500 models with 500 unique clustering solutions to classify the physiologic states of septic patients (Komorowski et al., 2018). This approach allowed another algorithm to learn associations among intravenous fluid doses, vasopressor doses, patient physiologic states, and patient outcomes to generate recommendations for resuscitation strategies. Subsequent analyses demonstrated that mortality was lowest when clinician actions aligned most closely with algorithm recommendations, suggesting opportunities to augment clinical decision-making.

Fuzzy clustering, a variation of centroid-based clustering, lets points belong to more than one cluster, offering potential advantages for clustering datasets that contain natural overlap among groups. Points are assigned to one or more clusters based on membership coefficients representing similarity to points in each cluster. Mathematically, this is accomplished by relaxing the constraint of assigning binary (yes or no) cluster membership. Instead, cluster membership values are assigned along a continuum from 0 (no) to 1 (yes). Biologically, this approach aligns with observations that boundaries between classes of patient and disease phenotypes are often indistinct. For example, there is substantial overlap in gene expression data across cancer types. Fuzzy clustering methods applied to gene expression data for leukemia, lymphoma, adenocarcinoma, and melanoma patients, along with dimensionality-reduction techniques, have demonstrated improved performance in associating genes with cancer types (Avogadri and Valentini, 2009). Fuzzy clustering is typically performed as a variant of centroid-based clustering, like *k*-means. As such, it shares the *k*-means disadvantages of sensitivity to outliers and requirement that investigators predetermine the number of clusters.

### Hierarchical clustering

Hierarchical methods use iterative merging of points based on pair-wise distances. In each step, the most similar points are merged into a single branch of a dendrogram. With each step, branches merge into progressively larger branches containing greater numbers of points, eventually forming a single branch containing all points. The dendrogram is then cut at a prescribed distance; cuts at longer distances result in fewer branches, or clusters. The prescribed distance can be determined by choosing a cut that (a) visually fits the natural distribution of the data, (b) optimizes cluster-wise distance metrics (e.g., Dunn's index), or (c) reflects underlying biology (e.g., a diagnostic threshold value). Hierarchical clustering methods have been used to identify groups of countries with similar labor market regulations, allowing analysis of important associations between socioeconomic conditions and public health that would remain hidden from traditional indicators like Gross National Product per capita (Muntaner et al., 2012). Visual interpretation of dendrograms and the absence of pre-specified cluster numbers facilitated these analyses; relative to other clustering methods, dendrograms are easy to interpret both conceptually and visually. Unlike centroid-based clustering, the number of clusters need not be assigned *a priori*; like centroid-based clustering, hierarchical clustering is sensitive to outliers. In addition, hierarchical clustering can be cumbersome for large datasets.

### Distribution-based clustering

Distribution-based methods assign points to clusters that have similar probability distributions for measures of center or spread like mean or variance. At the center of a cluster, the probability that a point belongs to that cluster is highest; with progressive distance from the cluster center, the probability of group membership decreases. For simulated data, this approach mimics the distribution sampling methods that generated the dataset and adapts well to natural distributions in the data. Therefore, Gaussian mixed models (GMM) are popular implementations of distribution-based clustering. In health care applications that use real, noisy data that do not fit Gaussian distributions, there is greater potential for overfitting (generating an algorithm that does not perform well on new data because it too closely reflects a limited training data set), especially when model complexity is unrestrained. Distribution-based clustering can adapt to non-Gaussian distributions, as previously demonstrated for associations between age and comorbidities, which do not follow normal distributions (Alhasoun et al., 2018).

### Density-based clustering

Density-based methods identify clusters as the densest regions in a data space that are separated from other clusters by low-density areas. The resulting cluster shapes adapt well to non-linear data. By design, outliers are not assigned to clusters. Therefore, unlike centroid-based and hierarchical clustering, density-based methods are insensitive to outliers. Outlier insensitivity offers unique advantages for clustering tasks related to complex pathophysiologic processes like neurodegenerative disease. The Density-Based Spatial Clustering of Applications with Noise (DBSCAN) algorithm identified noisy features outside cluster density boundaries in the more than 20,000 gene vectors that represent neurodegenerative disease-associated methylation processes (Mallik and Zhao, 2020). Removing noisy features allowed identification of 229 differentially methylated genes associated with Alzheimer's disease, bringing focus and clarity to subsequent analyses. Yet, these potential advantages are realized only when density-based clustering approaches are well-matched with the input dataset. Dense areas in data space are difficult to identify in sparse data. Additionally, the density-based clustering algorithms tend to be complex, rendering them slow on large datasets. Finally, density-based clustering algorithms are particularly sensitive to hyperparameters (parameters whose value is set by the user), underscoring the importance of search methods that identify sets of hyperparameters yielding optimal performance.

### Supervised clustering

Supervised clustering, sometimes called constraint-based clustering, involves user input regarding cluster properties like number, size, dimensions, or elements. By imposing these constraints, users can ensure that clustering results incorporate prior knowledge of biology (Lee and Hemberg, 2019). For example, CellAssign (Zhang A. W. et al., 2019) and Garnett (Pliner et al., 2019) use lists of marker genes for each cluster to perform automated cell-type annotation based on single-cell RNA sequencing data, on a new set of cells. Garnett (Pliner et al., 2019) uses defined cell markers to form an immune cell type hierarchy, trains a classifier to identify sets of cell marker thresholds for each cell type, then classifies cells *via* hierarchical clustering. After training on mouse lung samples, this approach annotated new data from a human lung tumor. CellAssign (Zhang A. W. et al., 2019) uses raw expression count data for a cell population, along with a set of known marker genes mapped to cell types as inputs in a Bayesian model, to calculate the probability that a cell belongs to one of the groups represented by the marker gene mapping. Therefore, injecting human knowledge of biology can steer a clustering algorithm toward an intended output. However, if performed carelessly, injecting human knowledge can increase risk of overfitting by enforcing so many rules and constraints that the result resembles statistical approaches rather than an algorithm that learns from data.

### Spectral or graph-based clustering

Spectral or graph-based clustering uses standard clustering methods (e.g., *k*-means) on specialized vector types called eigenvectors or on densities within a matrix (rectangular array of data) that represents a graph (Zhong et al., 2015). While standard graph-based methods like STING (Statistical Information Grid) and CLIQUE (Clustering In QUEst) use cell densities for cluster assignments, spectral clustering requires derivation of eigenvectors that can perform dimensionality reduction, rendering spectral or graph-based clustering especially effective for high-dimensional data containing noise and outliers. This approach has shown efficacy in grouping similar medical codes into clinically relevant concepts (Zhang L. W. et al., 2019). Similarly, dimensionality reduction with latent class analysis followed by *k*-means clustering has shown efficacy in representing complex medical conditions like frailty, cardiovascular complications, and psychiatric illness (Grant et al., 2020). Realizing the potential advantages of spectral clustering requires advanced understanding of vector spaces and linear transformations, and methods may be difficult to interpret for many clinicians.

### Consensus clustering

Consensus clustering, sometimes called aggregated clustering or clustering ensembles, uses multiple clusterings derived from (a) different clustering algorithms, (b) multiple permutations of a single algorithm, or (c) multiple iterations of a single algorithm on subgroups of a dataset to derive one, final set of cluster assignments. Consensus clustering has the theoretical advantages of minimizing overfitting and optimizing stability of cluster assignments, as has been shown for hierarchical clustering on genomic datasets from disparate sources and for identifying subgroups of heterogeneous intensive care unit patients (Vranas et al., 2017; Hulot et al., 2020).

## How to choose a clustering approach

Health care datasets can contain natural groupings, like apples and oranges that may be placed in separate bushels, or can contain a single mass of data, like a pizza that may be partitioned (cut) into slices. The former implies utility for a bottom-up, non-partitioning approach in which objects are grouped with others that have similar characteristics; the latter implies utility for a top-down partitioning approach in which one large group is divided into subgroups. Natural groupings or single masses of data can be visualized by a density-based algorithm called ordering points to identify the clustering structure (OPTICS; Ankerst, 1999). This approach generates a reachability plot illustrating the inherent structure of data. A jagged reachability plot suggests natural groupings amenable to non-partitioning methods; a smooth reachability plot suggests a single mass of data amenable to partitioning (cutting) methods, sometimes referred to as centroid-based clustering (e.g., *k*-means). Alternatively, when the reproducibility plot is smooth, users may recognize that there are no physiologically significant subgroups, and the single mass of data should be analyzed as a single group.

Beyond the natural groupings in data that may be apparent with OPTICS clustering, one must understand and apply the known strengths and weaknesses of different clustering methods described above and listed in Table 1.

## Cluster validation

Clustering algorithms always return results, but those results may not be reproducible. To test the reproducibility of the clustering approach, it is necessary to perform validation, ideally on an independent, external dataset. Cluster validation should be considered an essential final step for phenotype clustering in health care. A substantial body of literature from Bezdek supports a three-step process of first determining the optimal number of clusters, then performing portioning, and then performing validation (Bezdek, 1973, 2013; Bezdek and Harris, 1978; Pal and Bezdek, 1995). For instances in which attempts at validation fail to reproduce in independent, external datasets, one may wish to determine whether the failure is attributable to overfitting or to different distributions and patterns within the underlying data, suggesting that phenotypes themselves vary between datasets.

## Potential disadvantages or harms of clustering

All clustering methods share the weakness of finding potential clusters even when natural clusters do not exist. This underscores the importance of applying data-driven techniques as well as clinical and statistical expertise to clustering analyses. We quote Preud'homme and colleagues (Preud'homme et al., 2021) as they discuss an extraordinary effort to provide data-driven guidance for selecting clustering methods for heterogeneous data: “Despite the immense progress enabled by artificial intelligence in recent years, human experience and intuition remain the best judge in cluster analysis.” When human experience and intuition are suboptimal or are bent to fit a hypothesis, clustering analyses can distract from underlying patterns in data rather than reveal them.

## Opportunities for clustering to enhance health care delivery and research

### Revealing obscured associations in disease pathophysiology and predicting treatment response

Clustering can be used to pursue a deeper understanding of disease pathophysiology by revealing obscured associations, especially for syndromes with substantial depth, breadth, and complexity for which broad disease classification systems sacrifice precision. Few oncologists would stage all solid tumors as metastatic or non-metastatic alone, undermining the precision of clinical and research efforts by omitting pathologic grade, depth and anatomic level of local invasion, regional lymph node status, and the presence of locally advanced disease. Yet, similarly broad, imprecise classification systems are still used for other complex diseases. For example, secondary analyses of several acute respiratory distress syndrome (ARDS) trials have consistently identified both a hyper-inflammatory ARDS phenotype, featuring greater levels of circulating inflammatory cytokines and incidence of shock, and a hypo-inflammatory phenotype, featuring a favorable prognosis (Calfee et al., 2014, 2018; Famous et al., 2017; Sinha et al., 2018). Importantly, hyper- and hypo-inflammatory ARDS phenotypes may have different responses to targeted treatments. ARDS phenotypes were identified by latent class analysis: a probabilistic, distribution-based classification method. Similar work has been performed with partitioning or centroid-based clustering.

Similarly, Seymour et al. (2019) applied consensus *k*-means clustering to 29 routine, clinical and laboratory variables among sepsis patients, identifying four distinct phenotypes with unique pathophysiologic biomarker signatures and outcomes. Recognizing that nearly all targeted sepsis treatments have failed, they applied the four sepsis phenotypes to data from three randomized controlled trials that evaluated the efficacy of a toll-like receptor 4 inhibitor, early goal-directed therapy, or activated protein C. In a series of *post-hoc* trial simulations, they varied the proportions of each phenotype, yielding significant differences in treatment benefits and harms. For example, the original ProCESS trial demonstrated 0% chance of benefit from early goal-directed therapy, 15% chance of harm, and 85% chance of having no effect (Pro et al., 2014). *Post-hoc* simulations by Seymour and colleagues demonstrated that if all ProCESS trial patients had the alpha phenotype, characterized by fewer laboratory measurement abnormalities and less organ dysfunction, then early goal-directed therapy would have a 35% chance of benefit and a 65% chance of no effect. In other simulations performed exclusively with the other three phenotypes (beta: older patients with more chronic illness and renal dysfunction; gamma: more inflammation and pulmonary dysfunction; delta: more liver dysfunction and septic shock), early goal-directed therapy had a 0% chance of benefit and a >60% chance of harm. Seymour and colleagues also performed simulations for the PROWESS trial comparing activated protein C and placebo for patients with severe sepsis, which reported an 82% chance of a positive effect for activated protein C (Bernard et al., 2001). Xigris®, a recombinant form of human activated protein C, received FDA approval after the PROWESS trial but was withdrawn from the market when subsequent randomized trials showed no benefit (Abraham et al., 2005; Ranieri et al., 2012). In the simulations performed by Seymour and colleagues, when the alpha phenotype was increased to compose the majority of the study population, 50% of the simulations showed no difference for activated protein C. Collectively, these findings suggest that clustering analyses can reveal obscured associations that may be important to underlying pathophysiology, especially for instances in which conventional analyses are designed and powered to detect differences between treatment groups, and not between pathophysiological subsets. Clustering with simulation has the potential to generate hypotheses about pathophysiological subsets that can be tested in subsequent trials.

### Clinical trial enrollment

Clustering has the potential to augment the process of identifying patients for clinical trial enrollment, addressing a major challenge in producing high-level evidence that evolves the standard of care. In one review of randomized trials published in prominent journals, approximately 60% failed to meet recruitment targets or required extended recruitment periods (Puffer and Torgerson, 2003). Publication bias may have caused many more randomized trials with suboptimal enrollment to fail to achieve worthwhile impact. Inadequate enrollment can render a study underpowered, conferring increased risk for type II errors (not detecting a significant difference when it is present) and increased costs and resource use for an extended recruitment period.

To optimize clinical trial enrollment, *a priori* inclusion criteria and real-time screening must be robust; both processes can be augmented by clustering. Secondary analyses of clinical trial data suggest that treatment effects vary substantially across patient phenotypes that can be readily identified by clustering methods (Calfee et al., 2014, 2018; Famous et al., 2017; Seymour et al., 2019). Before enrollment begins, it may be advantageous to assess treatment responses across phenotypes in existing retrospective or prospective observational data and use these results to sharpen inclusion criteria. Once enrollment begins, screening often depends on the vigilance of clinicians and research coordinators to review hundreds or thousands of health records and ascertain whether patients meet enrollment criteria. Screening for a cluster phenotype-based clinical trial could, in the future, be automated across all participating institutions that use compatible electronic health record data models and variable names, but only in the context of further refining emerging technologies for real-time machine learning applications using electronic health record data (Ren et al., 2022). To improve the generalizability of this approach, data can be mapped to interoperable data models such as the open-source OMOP (Observational Medical Outcomes Partnership) common data model.

## Conclusions

Clustering methods offer important opportunities to mine data to discover natural structures and patterns that represent complex human pathophysiology. To add value to clinical care and research, optimal clustering practices require a thorough understanding of how to process and optimize data, select features, weigh strengths and weaknesses of different clustering methods, select the optimal clustering method, and apply clustering methods to solve problems. All clustering methods share the weakness that they find clusters even when natural clusters do not exist, underscoring the importance of applying data-driven techniques like OPTICS alongside statistical knowledge and clinical expertise. Iterative processes for optimizing clustering parameters and critical analysis by clinician experts is necessary to improve the efficiency and impact of phenotype clustering in health care. Applied properly, patient and disease phenotype clustering can reveal obscured associations that can help clinicians understand disease pathophysiology, predict treatment response, and identify patients for clinical trial enrollment.

## Author contributions

TL, GU, and AB contributed to conceptual design. TL, BS, and JB contributed to the literature review. TL drafted the manuscript. All authors contributed to development and/or interpretation of the content and provided critical revisions. All authors contributed to the article and approved the submitted version.

## Funding

TL was supported by the National Institute of General Medical Sciences (NIGMS) of the National Institutes of Health under Award Number K23GM140268. PT was supported by K07 AG073468 and R01AG121647 from the National Institute on Aging (NIA). PR was supported by National Science Foundation CAREER award 1750192, P30AG028740, and R01AG05533 from the NIA, R21EB027344 from the National Institute of Biomedical Imaging and Bioengineering (NIBIB), and R01GM-110240 from the NIGMS, R01EB029699, and R01NS120924. AB was supported by R01GM110240 from the NIGMS and 1R21EB027344 from the NIBIB. This work was supported in part by the National Center for Advancing Translational Sciences and Clinical and Translational Sciences Award to the University of Florida UL1TR000064. Funding sources had no role in study design, in the collection, analysis, and interpretation of data, in the writing of the report, or in the decision to submit the paper for publication.

## Conflict of interest

The authors declare that the research was conducted in the absence of any commercial or financial relationships that could be construed as a potential conflict of interest.

## Publisher's note

All claims expressed in this article are solely those of the authors and do not necessarily represent those of their affiliated organizations, or those of the publisher, the editors and the reviewers. Any product that may be evaluated in this article, or claim that may be made by its manufacturer, is not guaranteed or endorsed by the publisher.

## Author disclaimer

The content is solely the responsibility of the authors and does not necessarily represent the official views of the National Institutes of Health.
